# Let's chat(GPT): Implementation of a ChatGPT-generated social determinants of health activity

**DOI:** 10.1016/j.rcsop.2024.100553

**Published:** 2025-05-06

**Authors:** Karl R. Kodweis, Theodore J. Cory, Elizabeth A. Hall, Christa M. George, Katherine L. March

**Affiliations:** The University of Tennessee Health Science Center College of Pharmacy, Memphis, TN, United States of America

**Keywords:** Artificial intelligence, ChatGPT, Social determinants of health, Pharmacy education, Team-based learning

## Abstract

**Background:**

Artificial intelligence (AI)- powered chatbots have provided some notable benefits for learners. Educators are beginning to explore their possible utility and find ways to leverage AI in their classrooms.

**Objective:**

This study aimed to evaluate the implementation of ChatGPT-generated social determinants of health (SDOH) activity in a team-based pharmacy education course.

**Methods:**

Instructors asked the software to generate a set of learning objectives, an in-class activity, assessment strategies, and summative assessments for a student's conceptual understanding of SDOH. During a required first-year, team-based pharmacy course at the University of Tennessee Health Science Center, participants (*n* = 95) completed a ChatGPT-generated, in-class activity on SDOH within groups. The students' views on the quality of the activity were evaluated using five Likert-scale questions. Four of the questions assessed the applicability and usefulness of the assignment, with rankings on a scale of 1–4 (1 = strongly disagree; 4 = strongly agree). The fifth question evaluated the quality of the activity compared to activities generated by the instructor, using a scale of 1–5 (1 = far worse; 5 = far better).

**Results:**

For applicability and usefulness,”94.7 % (*n* = 90) of students agreed that “This in-class exercise was valuable to my professional development as a pharmacist;” 96.8 % (*n* = 92) agreed with “It is necessary for pharmacists to understand SDOH;” 94.7 % (n = 90) agreement with the statement, “*The quality of this in-class activity was on-par with other in-class activities in the course*;” and 90.5 % (*n* = 86) agreed with “*This in-class exercise was just as impactful to my professional development as other in-class activities.*” The majority of students (63.2 %; *n* = 60) selected either “somewhat better” (40 %; *n* = 38) or far better (23.2 %; *n* = 22) for, “Regarding quality, I feel the in-class activity was ____ than other in-class activities.”

**Conclusions:**

Most students reported that the ChatGPT-generated activity on social determinants of health was useful, applicable, and somewhat or far better than instructor activities. However, AI can generate incorrect information and potentially hinder student learning of conceptual frameworks; thus, instructors should review all output carefully.

## 1 Introduction

Artificial intelligence (AI) is a term for computer systems developed to imitate human intelligence.^1^^,^^2^ These computer systems can perform tasks that typically require human cognition, such as perception, comprehension, reasoning, learning, planning, and problem-solving.^2^^,^^3^ These technologies have countless uses, one of which is through AI-assisted chatbots. Historically, chatbots combine AI and natural language processing to provide human communication and interaction when engaged.^4^ ChatGPT (OpenAI, San Francisco, CA, USA) is an AI chatbot currently freely accessible to the public.5., 6, 7 When a prompt or query is input into the program, ChatGPT scours thousands of internet sources, automatically generating a response without requiring further input from the user.^7^

Given the sophisticated nature and capabilities of the program, ChatGPT has been used to write essays and scholarly papers and even take doctoral-level exams.^7^^,^^8^ This practice has led many educators and education systems to ban this technology.^9^^,^^10^ While educators debate the regulation and ethical ramifications of ChatGPT, adopting and implementing such technology is already underway. In 2022, IBM reported that AI utilization in businesses globally had reached 35 %, a noted 4 % increase from 2021.^11^

Educators are tasked with discovering positive ways to embrace new technologies in education.^12^^,^^13^ AI-powered chatbots have provided notable benefits for learners, including increased learning satisfaction, personalized learning experiences, and deeper collaboration and teamwork.^4^^,^^14^, ^15^, ^16^, ^17^ In pharmacy education, for example, AI-powered chatbots can be utilized to create clinical cases. Instructors can generate AI responses to patient questions on clinical topics and then instruct students on how to assess these AI answers and address any deficiencies.^18^ Specifically, in this example, using AI in this manner also enhances students' understanding, use, evaluation, and ethical considerations of these programs, often referred to as *AI literacy*.^19^ Some have noted the benefits of AI programs for simulation-based pharmacy education, showing that it enhances students' “management” and “expression” skills when used with a standardized patient for an Objective Structured Clinical Examination (OSCE).^20^

In this study, a ChatGPT-generated, in-class activity on the social determinants of health (SDOH) was developed and implemented, and student perceptions of the activity were evaluated.

## 2 Material and methods

### 2.1 Activity development

ChatGPT was used to create an activity for first-year pharmacy students in a skills-based pharmacy course, “Interprofessional Education and Clinical Simulation II” (IPECS II) at The University of Tennessee Health Science Center College of Pharmacy (UTHSC COP). The ChatGPT inputs and outputs can be found in Appendix A. ChatGPT was utilized to generate learning objectives, the group activity, assessment strategies for the educational outcomes, validated tools for SDOH assessment, and SDOH assessment verification. We decided to use the publicly available version of ChatGPT “as-is” to assess its ability to accurately generate educational content on this topic. Thus, we didn't provide any specific materials for the chatbot to learn from, asking it to rely on the most updated information available within the system.The group activity could not be developed after the initial input prompt alone; because of the “one-day long” verbiage used in the inputted prompt, the chatbot generated an activity designed to take place over 8 h. The instructors took this 8-h session and redesigned it to fit into the 2-h session that the class was scheduled for to ensure successful activity implementation.

Instructors also asked ChatGPT how to assess student learning, as shown in Appendix A. The content generated identified several practical methods to assess student understanding of the material. The assessment strategies ranged from formative and summative methods to self-reflection.

Lastly, ChatGPT was utilized to identify validated tools to assess student understanding of SDOH but was unsuccessful in doing so. The chatbot provided five scales to use, which stated they were “…validated for use in multiple countries and languages.” Upon investigation of the literature surrounding the suggested validated tools, all efforts were futile; none of the five recommended tools existed in the current literature. To ensure that the study investigator's search methods were not lacking, the chatbot was asked for specifics and was not able to provide those.

### 2.2 Activity implementation

Appendix B includes the final version of the ChatGPT-generated in-class activity. Participants (*n* = 95) completed the ChatGPT-designed SDOH activity within their pre-defined groups (groups assigned by the instructor) of 5–6 students, for a total of 16 groups. Students were not aware that this was a ChatGPT-generated activity.

During the first hour of the activity, each student group was assigned an SDOH domain and subdomain to research and was tasked with creating 2–3 presentation slides. Slides were required to include a definition of the SDOH domain/subdomain, impacts on health outcomes, real-life examples of how it affects individuals and communities, and potential solutions for pharmacists to address the issue. When complete, each group uploaded their submission to a cloud-based portal where presentations were divided into five domain-specific groups: economic stability, education access and quality, health care access and quality, neighborhood and built environment, and social and community context. Once all groups were finished and slides were uploaded, each group selected a representative to present their findings to the class.

The second hour of the activity included student-led presentations and open discussions for each domain/subdomain assigned. Each representative provided a 3- to 5-min presentation of their group's findings.

### 2.3 Data collection and analysis

The students' feedback for this activity was obtained through a concise 5-question survey featuring Likert-scale questions. All data was gathered using Qualtrics (Provo, UT) and analyzed using IBM SPSS Statistics for Mac (version 27, Chicago, IL). The students' perspectives on the quality of the activity were measured using five Likert-scale questions. Four of the questions assessed the relevance and usefulness of the assignment, with ratings on a scale of 1–4 (1 = strongly disagree; 4 = strongly agree) based on the following statements: “1) This in-class exercise was valuable to my professional development as a pharmacist; 2) It is necessary for pharmacists to understand SDOH; 3) The quality of this in-class activity was on par with other in-class activities in the course; 4) This in-class exercise was just as impactful to my professional development as other in-class activities.” The fifth question evaluated the quality of the activity compared to activities generated by the instructor, using a scale of 1–5 (1 = far worse; 5 = far better)for the statement, “Regarding quality, I feel the in-class activity was ____ than other in-class activities.” Descriptive statistics, including frequencies, means, and standard deviations, were computed for all Likert-type questions.

## 3 Results

The overall response rate for the survey was 100 % (*n* = 95). Most students agreed with all of the Likert statements assessing applicability and usefulness. 94.7 % (*n* = 90) of students agreed that “*This in-class exercise was valuable to my professional development as a pharmacist*;” 96.8 % (*n* = 92) agreed with “*It is necessary for pharmacists to* *understand SDOH*;” 94.7 % (n = 90) agreement with the statement, “*The quality of this in-class activity was on-par with other in-class activities in the course*;” and 90.5 % (*n* = 86) agreed with “*This in-class exercise was just as impactful to my professional development as other in-class activities.*” A breakdown can be seen in Table 1.

**Table 1:** Student perceptions of ChatGPT-generated activity with a 4-point Likert scale (1 = Strongly disagree, 4 = Strongly agree).

	P1 Students (n = 95)				
Item	Overall *Mean (SD)*	Strongly disagree *n, (%)*	Disagree *n, (%)*	Agree *n, (%)*	Strongly agree *n, (%)*
This in-class exercise was valuable to my professional development as a pharmacist.	3.3 (0.7)	3 (3.2)	2 (2.1)	52 (54.7)	38 (40.0)
It is necessary for pharmacists to understand SDOH.⁎	3.7 (0.6)	3 (3.2)	0 (0.0)	18 (18.9)	74 (77.9)
The quality of this in-class activity was on-par with other in-class activities in the course.	3.4 (0.7)	2 (2.1)	4 (4.2)	44 (46.3)	45 (47.4)
This in-class exercise was just as impactful to my professional development as other in-class activities	3.4 (0.8)	5 (5.3)	4 (4.2)	38 (40.0)	48 (50.5)

⁎ SDOH – Social determinants of health*.*

For quality, the majority of students (63.2 %; *n* = 60) selected either “*somewhat better*” (40 %; *n* = 38) or “*far better*” (23.2 %; *n* = 22) for “*Regarding quality, I feel the in-class activity was ____ than other in-class activities*.” A breakdown can be seen in Table 2.

**Table 2:** Student perceptions of ChatGPT-generated activity and comparison to other in-class activities, assessed with a 5-point quality scale.

	P1 Students (n = 95)				
Item	Far worse *n, (%)*	Somewhat worse *n, (%)*	Similar *n, (%)*	Somewhat better *n, (%)*	Far better *n, (%)*
Regarding quality, I feel the in-class activity was ____ than other in-class activities.	1 (1.1)	5 (5.3)	29 (30.5)	38 (40.0)	22 (23.2)

Students were also asked to list something they learned during the class session. Some students reported that researching the topic and presenting their findings to the entire class was engaging because it allowed them to learn from each other rather than just from the instructor. Some students reported learning new information about SDOH, such as the effects of air and water quality on patients' health. Other students reported learning ways besides medication management that pharmacists can affect SDOH.

## 4 Discussion

In pharmacy education, there have been recent discussions regarding the value of AI chatbots. The increasing use of AI in pharmacy practice and education necessitates a more formalized approach to AI instruction within pharmacy education.^18^^,^^21^ (Cain et al. and Abdel et. al). Current research primarily focuses on student satisfaction, yet there is a notable gap in evaluating the impact of AI chatbots on knowledge, skills, educational outcomes, and faculty workload. As a result, professional pharmacy programs must address the availability of tools, implementation challenges, and methods to enhance academic outcomes. ^18^^,^^21^ Implementing this AI-generated activity showed advantages and disadvantages compared to traditional classroom practices. The time associated with creating new course content for an active learning session can vary from a few hours to several days, depending on content complexity, class size, educator experience, and resources available. Content creation can be laborious for educators, especially for new or junior faculty or those needing to revise a course. For the IPECS II course at UTHSC COP, course directors are responsible for creating in-class, team-based learning activities for students to engage with for 2-h each week. This equates to 12 activities/semester, totaling approximately 24 h of in-class course content. While median creation time may vary between concepts and disciplines, the amount of outside-the-classroom time associated with generating these items can be substantial. Using ChatGPT, an in-class activity was entirely generated, revised for accuracy, and ready to implement in 2–3 h. Reducing class preparation time has the potential to have meaningful impacts on productivity by allowing faculty to allocate time to other scholarly endeavors.

Another advantage was that the activity was well-received by the students. Most students (93.5 %) found the activity valuable and of similar or better quality than other in-class activities performed during the course. Students found the activity to be engaging and enjoyed learning from each other. Many students reported learning new information about SDOH and how pharmacists can impact SDOH when caring for patients.

When using ChatGPT to create classroom content, it is crucial to remember that the premise of ChatGPT is to generate material. As noted above, when prompted for “*Validated tools to assess student understanding of social determinants of health,*” ChatGPT generated several false SDOH assessment scales with erroneous references. Because no validated tools exist to assess SDOH, the program generated a response, but it created invalid, non-existent content to meet the needs of the prompt entered. ChatGPT's capacity to generate non-existent information, often called “hallucinations,” poses a significant challenge. These hallucinations arise from algorithmic limitations and training data constraints, leading the chatbot to produce seemingly valid but ultimately inaccurate information.^22^^,^^23^ An alternative term, “confabulations,” coined by Hatem et al., more accurately describes this phenomenon as a fabrication within the data.^24^ Thus, cautious use and fact-checking are essential when relying on chatbots for information.^1^ This highlights the necessity of a critical mindset for reviewing any AI-generated materials. To the general population, these programs can offer valuable insight and information; however, for educators responsible for preparing students with accurate and up-to-date resources, the strict reliance on ChatGPT-generated materials is not recommended. Furthermore, educators should assess all items' accuracy, utility, and applicability to a student's development and learning goals for the course.

It is also imperative for the user to understand the limitations of using ChatGPT to generate learning-based outputs (i.e., create, design, write, assess). In its current version, the primary function of ChatGPT is to create the users desired content in a conversational, easy-to-follow format. ChatGPT was an effective tool when directly asked to perform a task or function like creating or designing a lesson plan. When a prompt was formatted as a question or inquiry, it returned information to achieve the goal of answering the question, regardless of the accuracy or correct information. ChatGPT answered the question asked but generated false content on information or tools that do not exist. Perhaps, even more concerningly, the generated tools were stated to be “*validated for use in multiple countries and languages*” (Appendix A).

The ethical and safe use of AI-powered tools is of utmost importance, especially in healthcare, where models must accurately capture causal relationships. However, significant concerns surround AI and deep learning model research, including the frameworks used to generate content. Recent discussions surrounding AI integration in medical education highlight the potential for deep learning systems to make accurate predictions but also note the challenges in understanding how they arrive at these decisions, often referred to as a “black box.”^25^ These concerns are particularly relevant in healthcare education, where a deep understanding of complex concepts is critical. To promote accountability and ensure that machine learning systems are not used to interfere with stakeholder autonomy in medicine arbitrarily, regulatory practices should establish procedures that limit their use to specific tasks that have been empirically validated for accuracy and reliability. This promotes accountability and freedom from domination by enabling stakeholders to use AI systems for tasks where they are most effective, even if the reasons for their superior performance remain unclear. Validation of system performance on multiple data sets that reflect real-world scenarios is necessary. Therefore, when machine learning systems perform decision tasks, the accuracy and reliability of humans and machines should be evaluated in empirical studies. Addressing these concerns and ensuring that AI-powered tools are used responsibly and transparently is essential.^18^^,^^25^, ^26^, ^27^

There are concerns that AI will make some jobs or professions obsolete. In this particular case, this is not yet a considerable threat, given that the current technology still requires professorial oversight and close review; however, as technology continues to advance, this may be a valid concern in the future.

From this experience, we observed various advantages and disadvantages of ChatGPT in a first-year pharmacy classroom. While these findings are useful for educators in this space, it should be noted that commercialized consumer AI programs are relatively new, and the technology is constantly evolving. These programs utilize machine-learning algorithms and continually find ways to improve efficiency, which means their intelligence and capabilities are constantly improving and becoming more advanced. Therefore, the disadvantages noted in this paper may be nullified with other AI-based programs/software or as newer, updated versions of these chatbots are released to the public.

## 5 Conclusion

ChatGPT has the potential to be a valuable tool for decreasing the class preparation time necessary without sacrificing the quality of instructor-generated content. Based on student perceptions, the ChatGPT-generated activity on SDOH was on par or better than other in-class activities from the same class. Some limitations of this software were noted, including generating incorrect and false information. Educators can benefit from utilizing this technology in their classrooms, and it is advised to extensively review the generated content and tailor any activities to meet the needs and requirements of the class.

## Generative artificial intelligence disclaimer

We want to emphasize that the authors have thoroughly evaluated, investigated and synthesized the content and discussions in this manuscript. Concerning generative artificial intelligence in our analysis, we utilized ChatGPT to evaluate and assess its effectiveness in creating educational materials and improving productivity in developing them. While we acknowledge the technology's contribution to the development of our in-class activity, we are aware of its potential risk of plagiarism. For the composition of the paper, generative intelligence, specifically Grammarly Premium v. 1.80.3.0, was not used for content creation but was employed to optimize the formatting, spelling, grammar, and overall tone. Nonetheless, all outputs generated in these capacities were thoroughly scrutinized for accuracy before incorporation into the manuscript. Therefore, please notify us immediately if you notice inappropriate citations or information.

## CRediT authorship contribution statement

**Karl R. Kodweis:** Data curation, Formal analysis, Investigation, Methodology, Project administration, Resources, Software, Supervision, Validation, Visualization, Writing – original draft, Writing – review & editing. **Theodore J. Cory:** Methodology, Writing – original draft. **Elizabeth A. Hall:** Writing – original draft. **Christa M. George:** Project administration, Writing – original draft. **Katherine L. March:** Conceptualization, Investigation, Methodology, Project administration, Validation, Writing – original draft, Writing – review & editing.

## Declaration of competing interest

The authors declare that they have no known competing financial interests or personal relationships that could have appeared to influence the work reported in this paper.

## Figures and Tables

**Table:** 

Learning Objectives **Prompt: Write 4 learning objectives for a class teaching about the social determinants of health** **Output:** • Describe the various social determinants of health and their impact on health outcomes. • Analyze how social, economic, and environmental factors influence health disparities. • Evaluate the role of policy and systemic factors in shaping health inequities. • Propose solutions and strategies for addressing the social determinants of health and promoting health equity.
**In-Class Activity** **Prompt: Design an innovative, one-day-long, team-based learning activity to teach first-year pharmacy students about the social determinants of health** **Output:** Here's an activity for teaching first-year pharmacy students about the social determinants of health: • Title: “Building Healthy Communities” • Duration: 1 day • Number of participants: 15–20 students, divided into 5–6 teams • Objective: To raise awareness and understanding of the social determinants of health and how they impact individuals and communities. • Materials: Flip charts, markers, and sticky notes; Access to the internet for research; Video-conferencing platform (e.g., Zoom) • Introduction (30 min) ◦ Start with a brief overview of the social determinants of health and why they are important to understand for future pharmacists. ◦ Explain the objective and format of the activity. ◦ Divide the students into teams and assign each team a specific social determinant of health to focus on (e.g., education, income, race/ethnicity, etc.). • Research and Presentation Preparation (2 h) ◦ Teams research their assigned social determinant of health and create a presentation to share with the rest of the class. ◦ The presentation should include: ▪ A definition of the social determinant of health ▪ Its impact on health outcomes ▪ Real-life examples of how it affects individuals and communities ▪ Potential solutions to address the issue • Presentations (1 h) ◦ Teams present their findings to the rest of the class via video-conferencing platform. ◦ Encourage the other teams to ask questions and engage in discussion. • Group Discussion and Reflection (45 min) ◦ Facilitate a group discussion to reflect on the presentations and encourage students to think about how their future role as pharmacists can contribute to addressing social determinants of health. ◦ Use sticky notes to write down key takeaways from the discussion and display them on a flip chart. • Conclusion (15 min) o Summarize the key points covered during the activity. o Highlight the importance of understanding and addressing the social determinants of health in their future careers as pharmacists. • Evaluation: ◦ Provide students with a feedback form to gather their thoughts on the activity and their overall learning experience. ◦ Use the feedback form to improve the activity for future classes.
**Assessing Educational Outcomes** **Prompt: How would you assess students meet the educational outcomes?** **Output:** To assess whether students have met the educational outcomes of the activity, you can use the following methods: • Formative assessment: ◦ Ask questions during the presentations and group discussions to check students' understanding of the social determinants of health and their impact on health outcomes. ◦ Encourage students to ask questions, share their opinions, and participate in discussions to assess their engagement and understanding. • Summative assessment: ◦ At the end of the activity, provide students with a written assessment that measures their knowledge of the social determinants of health and their potential impact on health outcomes. ◦ The assessment can include multiple-choice questions, short-answer questions, and essay questions that assess students' understanding of the topic and their ability to apply their learning to real-life situations. • Self-reflection: o Encourage students to reflect on their learning and the impact of the activity on their understanding of the social determinants of health. o Provide students with a self-reflection form that they can fill out at the end of the day to assess their own understanding of the topic and their overall learning experience. • Feedback: ◦ Encourage students to provide feedback on the activity and their learning experience. ◦ Use the feedback to continuously improve the activity and ensure that students are meeting the educational outcomes. Note: The assessment methods used should align with the activity's educational outcomes and objectives. Additionally, it's essential to provide students with constructive feedback and opportunities to reflect on their learning to help them grow and develop.
**Validated Tools for Assessment** **Prompt: What are validated tools to assess student understanding of social determinants of health?** **Output:** There are several validated tools that can be used to assess student understanding of social determinants of health, including: • Social Determinants of Health Awareness Scale (SDHAS): This is a 15-item self-reported questionnaire designed to assess awareness and understanding of the social determinants of health among healthcare professionals. It has been validated for use in multiple countries and languages. • Health Beliefs and Social Determinants of Health Knowledge Scale (HBSDK): This is a 16-item self-reported questionnaire designed to assess knowledge of the social determinants of health and beliefs about the impact of social factors on health outcomes. It has been validated for use in multiple countries and languages. • Social Determinants of Health Knowledge Test (SDHKT): This is a 25-item multiple-choice questionnaire designed to assess knowledge of the social determinants of health and their impact on health outcomes. It has been validated for use in multiple countries and languages. • Social Determinants of Health Literacy Scale (SDHLS): This is a 6-item self-reported questionnaire designed to assess knowledge and understanding of the social determinants of health among healthcare professionals. It has been validated for use in multiple countries and languages. • Social Determinants of Health Attitude Scale (SDHAS): This is a 12-item self-reported questionnaire designed to assess attitudes towards the social determinants of health and their impact on health outcomes. It has been validated for use in multiple countries and languages. These tools can be used to assess student understanding of the social determinants of health before and after a learning activity, and can provide valuable information on the effectiveness of the activity in improving student knowledge and understanding of the topic. Note: It's important to choose a validated tool that is culturally appropriate and relevant to the population being studied, and to use it appropriately, following the guidelines and recommendations of the tool developers.
**Assessment Tool Validity and Reliability** **Prompt: What are the 15-items used in the SDHAS?** **Output:** I'm sorry, but I don't have a specific list of the 15 items used in the Social Determinants of Health Awareness Scale (SDHAS) as the items may vary depending on the version of the scale and the population being studied. The SDHAS is a self-reported questionnaire designed to assess awareness and understanding of the social determinants of health among healthcare professionals. The items may include questions about: • Definition and understanding of social determinants of health • Knowledge of the major categories of social determinants of health (e.g. economic, environmental, and social factors) • Awareness of the impact of social determinants of health on health outcomes • Understanding of the role of various actors (e.g. government, health systems, and community organizations) in addressing social determinants of health • Attitudes towards the importance of addressing social determinants of health in healthcare These items are used to assess healthcare professionals' understanding of the social determinants of health and their impact on health outcomes. The scale may be adapted to meet the specific needs of different populations and settings. Note: If you need more information on the SDHAS, I recommend reaching out to the scale developers or the authors of the studies in which the scale was used. They may be able to provide you with more information on the specific items used in the scale.
**Generative Artificial Intelligence Disclaimer:** We would like to emphasize that the content and discussions in this manuscript have been thoroughly evaluated, investigated, and synthesized by the authors. Concerning generative artificial intelligence in our educational setting, we utilized ChatGPT to evaluate and assess its effectiveness in creating educational materials and improving productivity in developing them. While we acknowledge the technology's contribution to the development of our in-class activity, we are aware of its potential risk of plagiarism. Therefore, we kindly request that you notify us immediately should you notice any inappropriate citations or lack thereof.

**Table:** 

SDOH Domain	Sub-domains
Economic Stability	Poverty, Employment, Food Security, Transportation, Income
Education Access and Quality	Education, Early Childhood Development
Neighborhood and Built Environment	Access to Healthcare, Stress, Lack of Insurance/Uninsured
Healthcare Quality and Access	Housing, Water Quality, Neighborhood Environment, Air Quality
Social and Community Context	Racism, Bigotry, Social Support

## Appendix B ChatGPT-generated, faculty-edited activity for the SDOH

**Learning Objectives:** By the end of this session, you should be able to:

- Describe the various social determinants of health and their impact on health outcomes. Analyze how social, economic, and environmental factors influence health disparities.

- Evaluate the role of systemic factors in shaping health inequities.

- Propose solutions and strategies for addressing the social determinants of health and promoting health equity.

**Instructions:** Each team will be assigned a social determinant of health (SDOH) domain and subdomain for the class session from the available options (in the table below). This activity will comprise 2-part: a Research/Presentation Preparation and a Class Presentation.

**Part 1 - Research/Presentation Preparation (20** min**).**

Teams research their assigned social determinant of health and create a brief presentation to share with the rest of the class. Each group will be asked to upload 2–3 PowerPoint slides to a domain-specific, shared-online PowerPoint document. The presentation should include the following:

- A definition of the SDOH domain and subdomain

- Its impact on health outcome

- Real-life examples of how it affects individuals and communities

- Potential solutions to address the issue, as a pharmacist

**Part 2 - Class Presentation (60** min**).**

Each team will select one (1) representative from their group to present the findings and concepts related to the assigned domain/subdomain. Each domain is allotted 10 min for the presentation and 2 min in between each for transitions. Each SDOH domain will have 3–4 subdomains and 3–4 presenters, requiring the time allotment to be evenly distributed between presenters.

**Generative Artificial Intelligence Disclaimer:** We would like to emphasize that the content and discussions in this manuscript have been thoroughly evaluated, investigated, and synthesized by the authors. Concerning generative artificial intelligence in our educational setting, we utilized ChatGPT to evaluate and assess its effectiveness in creating educational materials and improving productivity in developing them. While we acknowledge the technology's contribution to the development of our in-class activity, we are aware of its potential risk of plagiarism. Therefore, we kindly request that you notify us immediately should you notice any inappropriate citations or lack thereof.
